# MicroRNA-33-5p inhibits cholesterol efflux in vascular endothelial cells by regulating citrate synthase and ATP-binding cassette transporter A1

**DOI:** 10.1186/s12872-021-02228-7

**Published:** 2021-09-13

**Authors:** Qiong Xie, Jianqiang Peng, Ying Guo, Feng Li

**Affiliations:** 1 Department of Cardiology, Hunan Provincial People’s Hospital, The First Hospital Affiliated With Hunan Normal University, Changsha, 410005 Hunan People’s Republic of China; 2 Departments of Cardiovascular Surgery, The Second Xiangya Hospital of Central South University, middle Ren-Min Road No. 139, Changsha, 410011 Hunan People’s Republic of China

**Keywords:** ox-LDL, Citrate synthase, ABCA1, miR-33-5p, Vascular endothelial cells

## Abstract

**Background:**

A high level of total cholesterol is associated with several lipid metabolism disorders, including atherosclerosis and cardiovascular diseases. ATP-binding cassette (ABC) transporter A1 (ABCA1) and miR-33-5p play crucial roles in atherosclerosis by controlling cholesterol efflux. While citrate is a precursor metabolite for lipid and cholesterol synthesis, little is known about the association between citrate synthase (CS) and cholesterol efflux. This study investigated the role of the miR-33-5p/ABCA1/CS axis in regulating cholesterol efflux in vascular endothelial cells (VECs).

**Materials and methods:**

VECs were treated with oxidized low-density lipoprotein cholesterol (ox-LDL), or pretreated with plasmids overexpressing CS, ABCA1, siRNAs against CS and ABCA1, and an miR-33-5p inhibitor. Cell apoptosis, cellular senescence-associated β-galactosidase activity, inflammation, and cholesterol efflux were detected.

**Results:**

Treatment with ox-LDL decreased ABCA1 and CS levels and increased miR-33-5p expression and apoptosis in dose-dependent manners. In contrast, treatment with the miR-33-5p inhibitor and ABCA1 and CS overexpression plasmids inhibited the above-mentioned ox-LDL-induced changes. In addition, treatment with ox-LDL decreased cholesterol efflux, induced aging, and promoted the production of inflammatory cytokines (i.e., IL-6 and tumor necrosis factor TNF-α), as well as the expression of Bax and Caspase 3 proteins in VECs. All these changes were rescued by miR-33-5p inhibition and ABCA1 and CS overexpression. The inhibition of ABCA1 and CS by siRNAs eliminated the effects mediated by the miR-33-5p inhibitor, and knockdown of CS eliminated the effects of ABCA1 on VECs.

**Conclusions:**

This study demonstrated the crucial roles played by the miR-33-5p/ABCA1/CS axis in regulating cholesterol efflux, inflammation, apoptosis, and aging in VECs, and also suggested the axis as a target for managing lipid metabolism disorders.

**Supplementary Information:**

The online version contains supplementary material available at 10.1186/s12872-021-02228-7.

## Introduction

Lipid metabolism disorders are associated with several diseases, including hyperlipidemia, atherosclerosis, coronary heart disease, and cardiovascular diseases (CVDs). Lipid metabolism disorders are characterized by increased levels of total cholesterol (TC) and low-density lipoprotein (LDL) cholesterol and decreased levels of high-density lipoprotein (HDL) cholesterol [1, 2]. In addition to dysregulation of lipid metabolism, aberrant inflammatory responses are common in the diseases mentioned above, and are leading causes of mortality resulting from CVD and atherosclerosis [2–4]. Low density lipoproteins (LDLs) are lipoprotein particles that carry cholesterol into peripheral tissue cells, and can become oxidized to form oxidized LDLs. When LDL levels (especially ox-LDLs) become excessive, the cholesterol they carry accumulates on the arterial wall and ultimately causes arteriosclerosis. Oxidized low-density lipoproteins (ox-LDLs) participate in the formation and progression of lesions by eliciting lipid accumulation, and causing inflammation and toxic events [5, 6]. It was reported that ox-LDLs combines with their major receptor (Lectin-like oxidized low-density lipoprotein receptor-1 [LOX-1]) to activate the NF-κB signaling pathway [7, 8]. Ox-LDLs also affect the generation and utilization of endothelium-derived nitric oxide (NO), impair endothelium-dependent vascular diastolic function, and initiate the formation and development of AS plaques [7, 9–11].

Atherosclerosis is characterized by the formation of lipid plaques which mainly consist of lipid-laden foam cells transferred from macrophages, and therefore decrease the uptake of LDLs from blood vessel walls [2, 12]. Several factors are associated with cholesterol efflux; these factors include the ATP-binding cassette (ABC) transporter A1 (ABCA1) protein family [13], miR-33-5p [14–16], inflammatory factors such as interleukin (IL)-6 and tumor necrosis factor (TNF)-α [17], and the 3-hydroxy-3-methylglutaryl coenzyme A reductase (HMGCR) enzyme. ABCA1 plays a crucial role in removing excess lipids by initiating reverse cholesterol transport (RCT) and mediating the transport of intracellular free cholesterol and phospholipids from peripheral tissues to lipid-poor apolipoprotein A I (ApoA1) [18]. In addition, ABCA1 also promotes the production of athero-protective HDL cholesterol [18, 19]. MiR-33-5p targets ABCA1, and knockdown of miR-33-5p decreases the levels of total cholesterol (TC) and TNF-α, and promotes cholesterol efflux from foam cells [13, 20]. It has been reported that the inflammatory cytokines IL-6 and TNF-α can suppress ABCA1 expression and thereby inhibit cholesterol efflux [17].

Citrate is a precursor metabolite for lipid and cholesterol synthesis, and accumulation of exogenous citrate promotes inflammation in visceral adipose tissue [21]. Lower rates of citrate synthase (CS) activity have been reported to be associated with increased levels of cleaved caspase-3 and lipotoxicity in C1C12 muscle cells [22]. Our previous experiments showed that CS is a direct target of miR-33-5p. However, there have been no reports of CS activity being associated with cholesterol efflux or lipid metabolism disorders such as atherosclerosis or CVD.

This current study investigated how the miR-33-5p/ABCA1/CS axis helps to control cholesterol efflux in vascular endothelial cells (VECs). VECs were treated with ox-LDLs and pretreated with a miR-33-5p inhibitor, ABCA1 and CS expression plasmids, or with siRNAs. The effects on cell apoptosis, cellular cholesterol efflux, and inflammatory cytokines (IL-6 and TNF-α) that occurred in response to gene modulation were detected. Our results provide novel insights into potential mechanisms associated with lipid metabolism.

## Materials and methods

### Cells, culture conditions, and treatments

Human VECs (CRL-1730, ATCC, Manassas, VA, USA) were maintained in DMEM (Hyclone, Logan, UT, USA) containing 1% antibiotics (penicillin–streptomycin), 10% FBS (Sigma-Aldrich; Merck KGaA, Darmstadt, Germany), with or without oxidized low-density lipoproteins (ox-LDLs) at a low (50 μg/mL), moderate (100 μg/mL) or high concentration (200 μg/mL) [23]. The ox-LDLs (Shanghai Jingke Chemical Technology Co., LTD, Shanghai, China) were dissolved in PBS, and then diluted to different concentrations. The VECs were incubated in DMEM at 37 °C in a 5% CO_2_ atmosphere, and then induced with ox-LDLs for 48 h.

### Plasmids and cell transfections

Plasmids overexpressing ABCA1 (OE-ABCA1) and CS (OE-CS) were constructed using the pcDNA3.1 vector (Genechem Co. Ltd., Shanghai, China) and full CDS sequence cloning. The polymerase chain reaction (PCR) was used to clone the ABCA1 or CS coding sequences into the plasmid pcDNA3.1 vector. The pcDNA3.1 vector without the *ABCA1* and *CS* genes served as a negative control. pcDNA3.1-ABCA1 or pcDNA3.1-CS was transfected into VECs (6-well plates, 1.0 × 10^6^/cm^2^). Short interfering RNAs (siRNAs) against human *ABCA1* and *CS* genes, miR-33-5p mimics (B01001), inhibitors (B03001) (chemically modified inhibitors that specifically targeted miR-33-5p), and scramble sequences were obtained from GenePharma Corporation (Shanghai, China). Cell transfections were performed using Lipofectamine 2000 (Invitrogen, Carlsbad, CA, USA) according to the manufacturer’s instructions. Cells transfected with the empty pcDNA3.1 vector or scrambled sequences served as negative controls (NCs) for overexpression and for siRNA, mimics or inhibitors, respectively. Cells were transfected with the different agents for 48 h before harvesting. Each experiment was repeated 3 times.

### Dual-luciferase reporter assay

Possible interactions between miR-33-5p and ABCA1 or CS were predicated using TargetScanHuman (http://www.targetscan.org/vert_72/), and subsequently verified by using the dual-luciferase reporter system. Luciferase vectors containing miR-33-5p binding sites (wild type, WT; and mutant, MUT, 7 base substitutions) in the 3′ UTR regions of *ABCA1* or *CS* genes were constructed using XhoI and NotI restriction enzymes; Thermo Fisher Scientific Inc., Waltham, MA, USA) and a psiCHECK-2 expression vector (Promega, Madison, WI, USA). Cell transfections were performed using Lipofectamine 2000 (Invitrogen).

### Enzyme-linked immuno sorbent assay (ELISA)

Cell culture samples were collected and prepared for ELISA. The levels of CS, interleukin-6 (IL-6), and tumor necrosis factor-α (TNF-α) in the samples were detected using ELISA kits (Elabscience Biotechnology Co. Ltd., Wuhan, China). A microplate reader (Thermo Fisher Scientific Inc.) was used to measure optical density (OD) at 450 nm.

### Cholesterol efflux determination

The total cholesterol efflux in culture medium and cell lysate was measured using a Cholesterol Efflux Fluorometric Assay Kit (BioVision, San Francisco, CA USA) according to the manufacturer’s protocol [24]. The fluorescence-labeled cholesterol was washed with 200 μL of phenol red-free, serum-free RPMI medium and then incubated in serum-free RPMI medium containing cholesterol acceptor ApoA 1 (50 μg/well) for 16 h at 37 °C. The total fluorescence intensity at 48 h post-transfection or treatment was detected at excitation and emission wavelengths of 482 nm and 515 nm, respectively. Cholesterol efflux was calculated as: 100% x media fluorescence intensity/total fluorescence intensity.

### Flow cytometric analysis and aging assay

Cell apoptosis was detected by flow cytometry. Cells treated or transfected with different agents for 48 h were harvested and fixed. Cell apoptosis was detected by using an Annexin V-FITC/PI Double Staining Kit (BD Biosciences, San Jose, CA, USA) according to the manufacturer’s instructions. The percentages of apoptotic VECs were analyzed using a BD FACS Calibur flow cytometer (BD Biosciences). Aged cells were detected by using a β-Galactosidase staining kit (Jiancheng Biological Company, Nanjing, China) according to the manufacturer's recommended protocol. Senescence-associated β-galactosidase (SA-β-Gal) in aged cells was detected as previously described [25]. Cellular images were captured using a microscope (Motic, Xiamen, China). Each experiment was repeated 3 times.

### Quantitative real-time PCR (qRT-PCR) analysis

Total cellular RNA was extracted using TRIzol reagent (Invitrogen), and first strand cDNA was synthesized using a Bestar qPCR RT Kit (DBI Bioscience, Shanghai, China). MiR-33-5p expression was achieved by reverse transcription that was performed using a stem-loop primer. A DBI Bestar ® SYBR Green qPCR master mix kit (DBI Bioscience) was used for qRT-PCR amplification conducted on an Mx3000P Agilent Stratagene PCR machine (Agilent, Santa Clara, CA, USA). The primers were synthesized by Sangon Biotechnology Co., Ltd. (Shanghai, China; Table 1). The following conditions were used for amplification: 95 °C for 2 min; followed by 40 cycles of 94 °C for 20 s, 58 °C for 20 s, and 72 °C for 20 s. The relative levels of mRNA or miR-33-5p expression were calculated using standard 2^−△△Ct^ methods. U6 and GAPDH served as internal reference genes.

**Table 1:** Primers used for quantitative real time PCR analysis

Gene	Primer sequence 5’-3’
Hsa-miR-33	F: ACACTCCAGCTGGGGTGCATTGTAGTTGCAT RT: CTCAACTGGTGTCGTGGAGTCGGCAATTCAGTTGAGTGCAATG
All	R: CTCAACTGGTGTCGTGGA
ABCA1	F: ACCCACCCTATGAACAACATGA R: GAGTCGGGTAACGGAAACAGG
CS	F: AACTGCTACCCAAGGCTAAGG R: CTTTTGAGAGCCAAGATACCTGT
HMGCR	F: AACTGCTACCCAAGGCTAAGG R: CTAAAATTGCCATTCCACGAGC
U6	F: CTCGCTTCGGCAGCACA R: AACGCTTCACGAATTTGCGT
GAPDH	F: TGTTCGTCATGGGTGTGAAC R: ATGGCATGGACTGTGGTCAT

*F* forward primer, *R* revise primer, *RT* reverse transcription

### Western blot analysis

The total cellular proteins were extracted using RIPA lysis buffer (Beyotime, Shanghai, China), and the protein concentration in each extract was determined using a protein quantification kit (Thermo Fisher Scientific). An aliquot of total protein from each extract was separated by 10% SDS-PAGE (Sangon Biotechnology Co., Ltd), and the protein bands were transferred onto PVDF membranes (Millipore Corporation, Burlington, MA, USA), which were subsequently blocked with 5% skim milk powder (Beyotime). Next, the membranes were incubated with specific primary antibodies against HMGCR (1: 1000; ab174830, Abcam, Cambridge, UK), ABCA1 (1: 1000; #96292, CST, Danvers, MA, USA), CS (1: 1000; #14309, CST), Caspase 3 (1: 1000; #14220, CST), Bax (1: 1000; #5023, CST), Bcl-2 (1: 1000; #4223, CST), and GAPDH (1: 2500; ab9485, Abcam), and subsequently incubated with a secondary antibody (HRP Goat anti-rabbit/mouse IgG, 1: 20,000; Boster, Wuhan, China). The immunostained protein bands were detected using enhanced chemiluminescence luminous fluid (Millipore), and band staining intensity was analyzed using a scanner (Microtek, Carson, CA, USA).

### Statistical analysis

The GraphPad Prism 8.0 (GraphPad, La Jolla, USA) was used for data analysis. Experiments were repeated in three times, all data are expressed as a mean value ± standard deviation (SD). The distribution normality of all data was verified prior before analysis by one-way ANOVA. Continuous data obtained from multiple groups was analyzed by one-way ANOVA followed by Tukey's post hoc test. A P-value < 0.05 was considered to be statistically significant, P-value < 0.01 was considered as obvious statistical significance.

## Results

### Ox-LDL increased miR-33-5p levels and decreased ABCA1 and CS levels

**Fig. 1:**
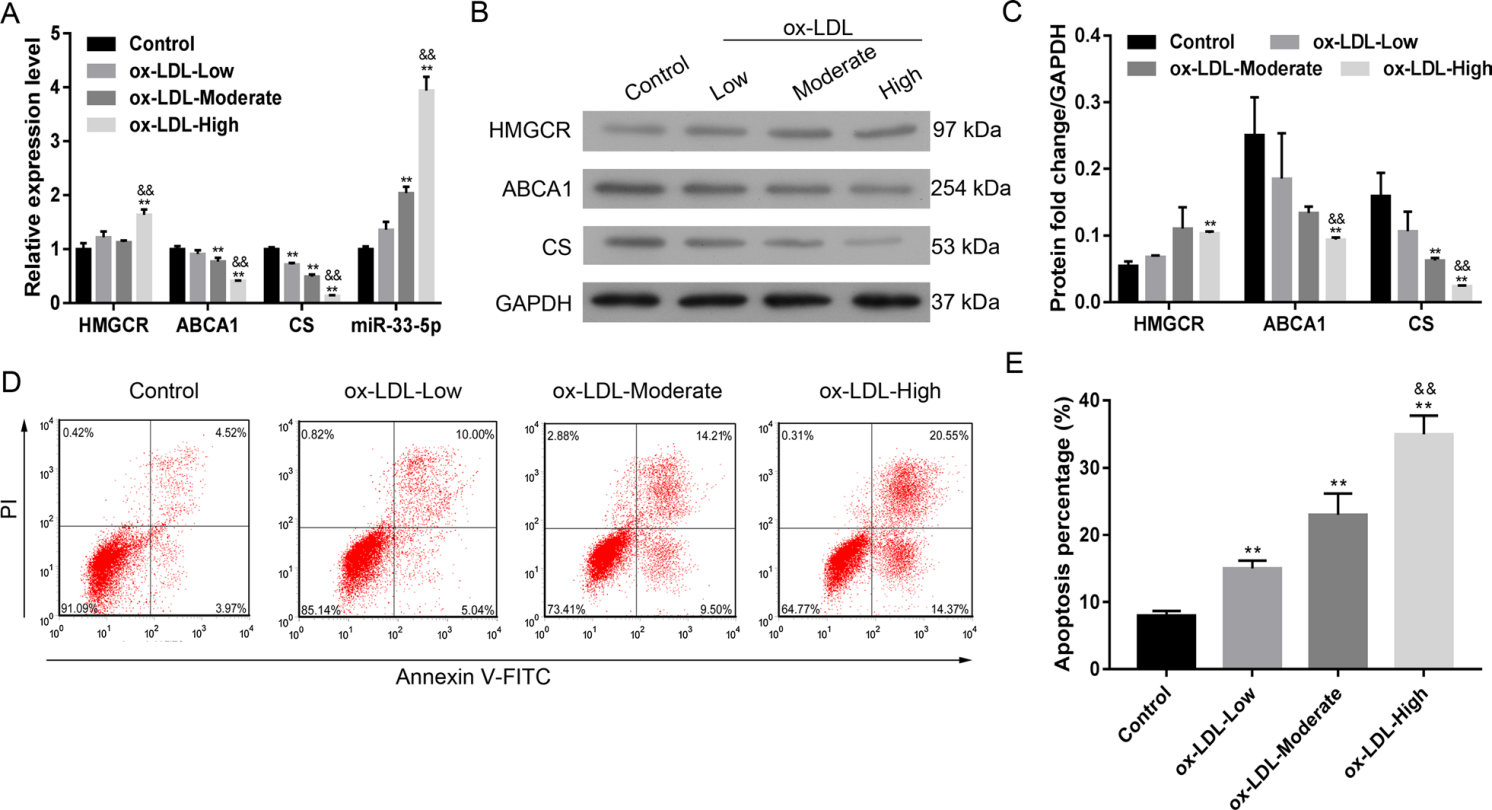
Effect of ox-LDL on VECs. **A** The levels of HMGCR, ABCA1, CS, and miR-33-5p mRNA expression in response to ox-LDL treatment. **B**, **C** the expression of HMGCR, ABCA1, and CS proteins in response to ox-LDL treatment. **D**, **E** the percentages of apoptotic VECs after ox-LDL treatment. Ox-LDL, oxidized low-density lipoprotein. Ox-LDL-low: 50 μg/mL; ox-LDL-moderate: 100 μg/mL; ox-LDL-high: 200 μg/mL. **p < 0.01 vs. control by one-way ANOVA followed by the Tukey’s post hoc test. ^&&^*p* < 0.01 vs. moderate by one-way ANOVA followed by the Tukey’s post hoc test.The above experiments were repeated three times and the mean value was obtained. The original bands of WB are presented in Additional file 1: [1A]

Administration of ox-LDL to VECs induced a dose-dependent inhibition of both ABCA1 and CS expression and increased miR-33-5p expression (Fig. 1A–C). HMGCR expression was only increased by the high concentration of ox-LDL. Ox-LDL also induced dose-dependent apoptosis in VECs (Fig. 1D, E). These data suggest that ox-LDL has a dose-dependent effect on both ABCA1 and CS expression, as well as on VEC apoptosis, whereas no such dose-dependent effects were found for HMGCR. Accordingly, we selected the moderate dose of ox-LDL for use in further experiments.

### Suppression of miR-33-5p impeded ox-LDL-induced changes in VECs

**Fig. 2:**
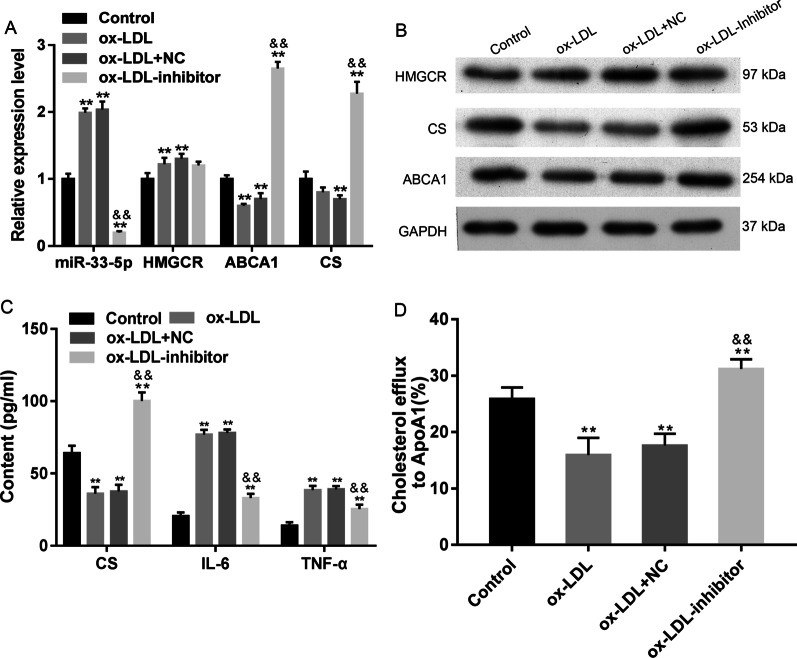
Effect of miR-33-5p inhibition on VECs. **A** the relative expression profiles of miR-33-5p, HMGCR, ABCA1, and CS mRNA. **B** The expression of HMGCR, ABCA1, and CS proteins in response to miR-33-5p inhibition. The original bands of WB are presented in Additional file 1: [1B]. **C** the levels of CS, IL-6, and TNF-α in VECs. **D** the cellular cholesterol efflux to ApoA 1 (50 μg/well) in VECs. Ox-LDL, oxidized low-density lipoprotein.NC, inhibitor negative control. **p < 0.01 vs. control by one-way ANOVA followed by Tukey's post hoc test. ^&&^*p* < 0.01 vs. ox-LDL by one-way ANOVA followed by Tukey's post hoc test. The above experiments were repeated three times and the mean value was obtained

**Fig. 3:**
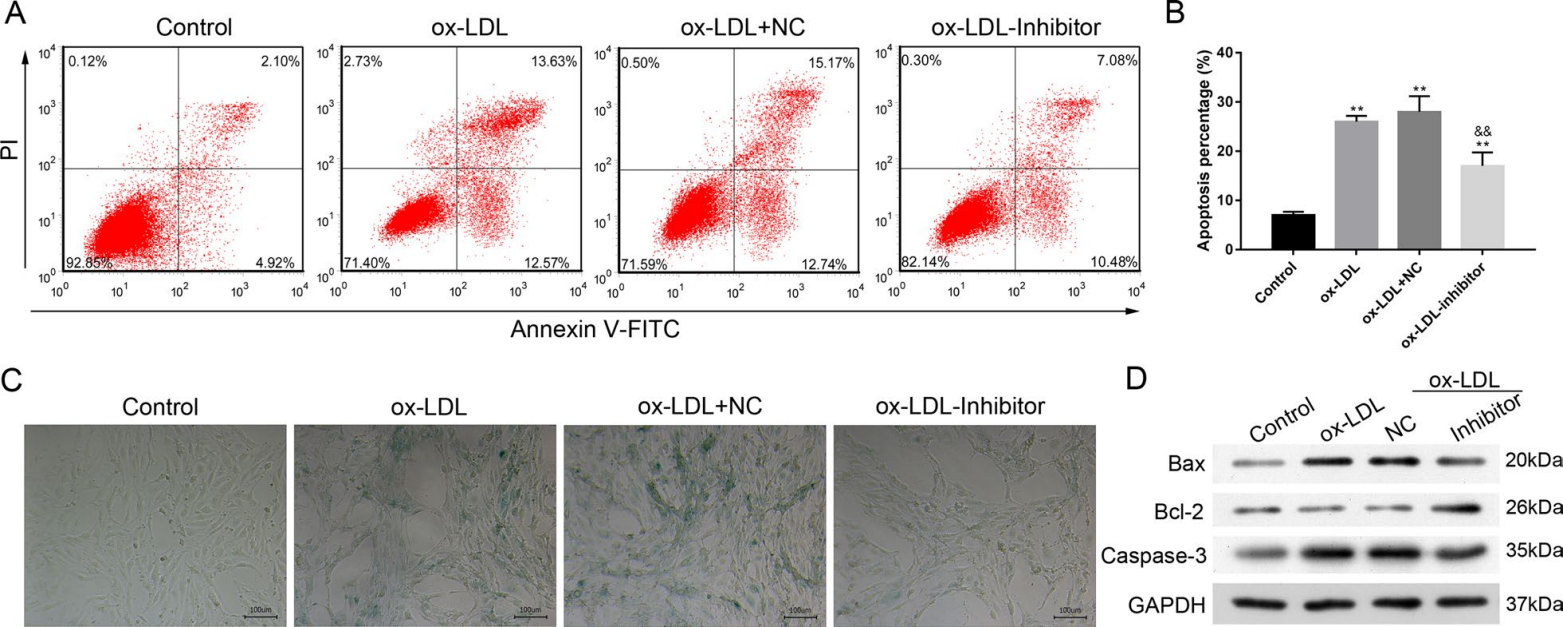
Influence of miR-33-5p inhibition on apoptosis and aging of VECs. **A**, **B** the percentages of apoptotic VECs. **C** The inhibitory effect of miR-33-5p on senescence-associated β-galactosidase (SA-β-gal) activity in VECs. The repeated results were shown in Aditional file 2: Supplementary Figure 3. **D** The expression of apoptosis-related proteins. Ox-LDL, oxidized low-density lipoprotein. NC, inhibitor negative control. The original bands of WB are presented in Additional file 1: [1C]. **p < 0.01 vs. control by one-way ANOVA followed by Tukey's post hoc test. ^&&^*p* < 0.01 vs. ox-LDL by one-way ANOVA followed by Tukey's post hoc test. The above experiments were repeated three times and the mean value was obtained

Inhibition of miR-33-5p impeded the ox-LDL-induced expression of miR-33-5p and HMGCR, and significantly increased the levels of both ABCA1 and CS expression (*p* < 0.01, Fig. 2A, B). The decreases in CS content and cholesterol efflux in VECs induced by ox-LDL were recovered by treatment with the miR-33-5p inhibitor (*p* < 0.01, Fig. 2C, D). The ox-LDL-induced increases in cellular IL-6 and TNF-α levels were attenuated by the miR-33-5p inhibitor (*p* < 0.01, Fig. 2C), as well as the levels of cell apoptosis and SA-β-gal activity (Fig. 3A–D). Bax and Caspase 3 expression were decreased by the miR-33-5p inhibitor, while Bcl-2 expression was upregulated by the miR-33-5p inhibitor (Fig. 3D). These data showed that miR-33-5p inhibition impeded ox-LDL-induced inflammation, apoptosis, and aging of VECs by increasing ABCA1 and CS expression and cholesterol efflux.

### ABCA1 and CS overexpression impeded ox-LDL-induced changes in VECs

**Fig. 4:**
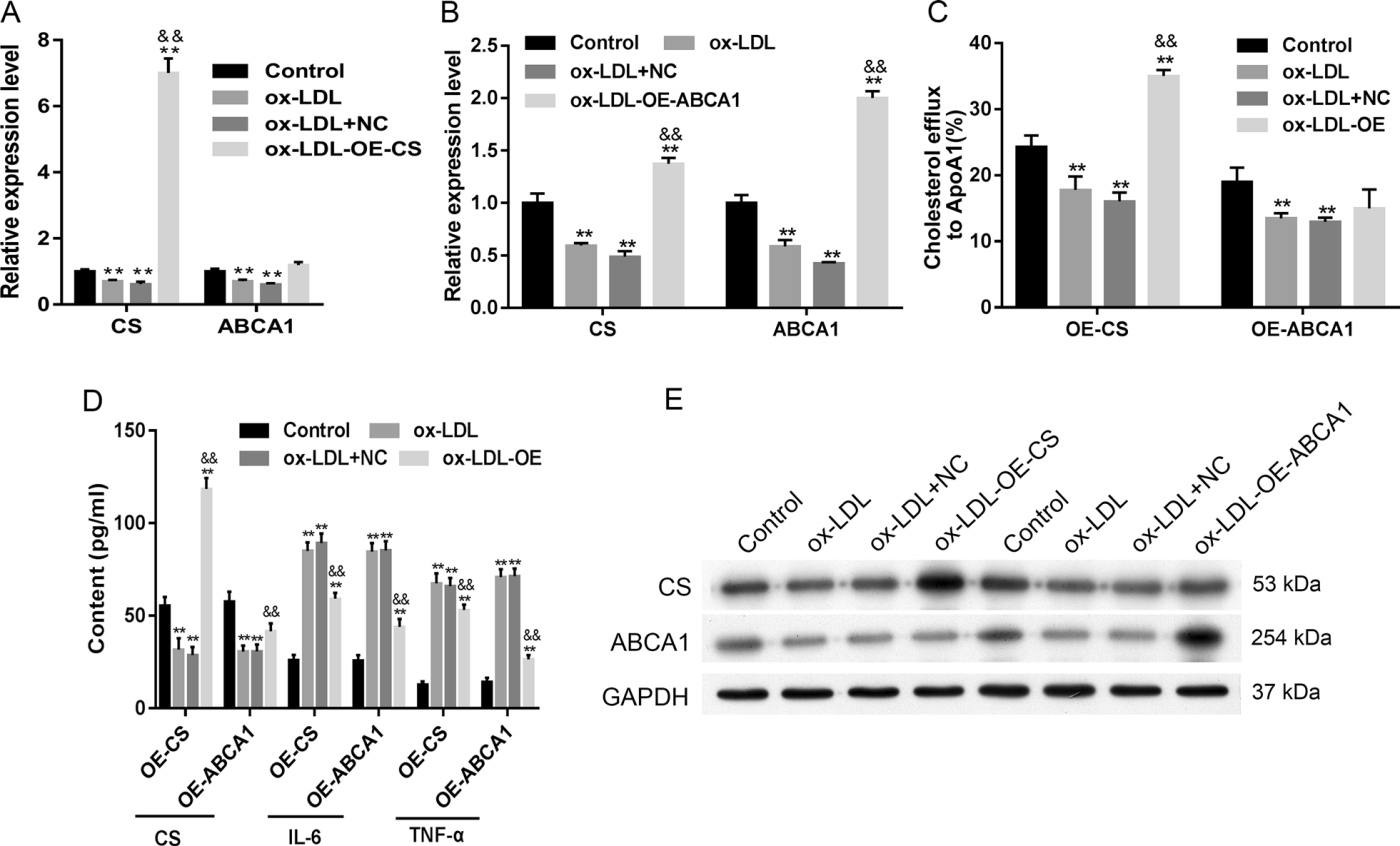
Effect of CS and ABCA1 overexpression on cellular secretion. **A**, **B** the overexpression of CS and ABCA1 impeded ox-LDL-reduced expression. **C** The cellular cholesterol efflux to ApoA 1 (50 μg/well) in VECs. **D** The levels of CS, IL-6, and TNF-α in VECs. **E** The levels of CS and ABCA1 protein expression in VECs. Ox-LDL, oxidized low-density lipoprotein. NC, overexpression negative control. The original bands of WB are presented in Additional file 1: [1D]. **p < 0.01 vs. control by one-way ANOVA followed by Tukey's post hoc test. ^&&^*p* < 0.01 vs. ox-LDL by one-way ANOVA followed by Tukey's post hoc test. The above experiments were repeated three times and the mean value was obtained

**Fig. 5:**
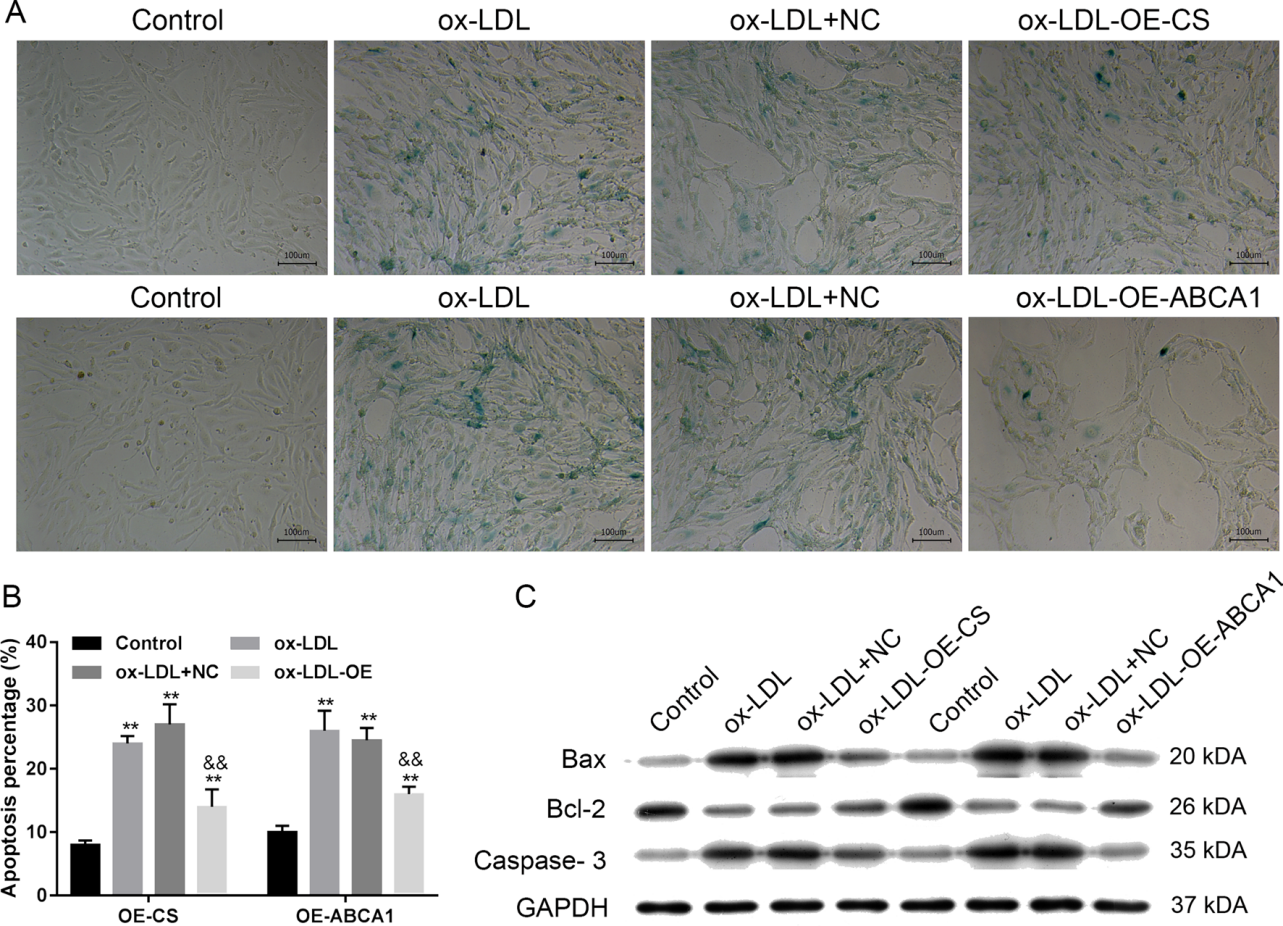
Effect of CS and ABCA1 overexpression on cellular apoptosis and aging. **A** the levels of senescence-associated β-galactosidase (SA-β-gal) activity in VECs. The repeated results were shown in Aditional file 2: Supplementary Figure 4. **B** the percentages of apoptotic VECs. **C** the expression of apoptosis-related proteins. Ox-LDL, oxidized low-density lipoprotein. NC, overexpression negative control. The original bands of WB are presented in Additional file 1: [1E]. **p < 0.01 vs. control by one-way ANOVA followed by Tukey's post hoc test. ^&&^*p* < 0.01 vs. ox-LDL by one-way ANOVA followed by Tukey's post hoc test. The above experiments were repeated three times and the mean value was obtained

We next examined whether overexpression of ABCA1 and CS in VECs would impede the ox-LDL-induced inhibition of ABCA1 and CS (Fig. 4A, B, E). Our data showed that overexpression of ABCA1 significantly increased both CS and ABCA1 levels, while overexpression of CS only significantly increased CS levels (*p* < 0.01, Fig. 4A, B). The decreases in CS content and cholesterol efflux, as well as the increases in IL-6 and TNF-α levels in VECs induced by ox-LDL were rescued by ABCA1 and CS overexpression (*p* < 0.01, Fig. 4B, C). Both ABCA1 and CS expression, and especially the former, decreased ox-LDL-induced cell apoptosis and SA-β-gal activity (Fig. 5A, B). Cellular SA-β-gal activity, apoptosis, and Bax and Caspase 3 expression were all increased by ox-LDL administration and rescued by ABCA1 and CS expression (Figs. 4, 5), while Bcl-2 expression was decreased by ox-LDL administration and rescued by ABCA1 and CS expression (Fig. 5D).

### MiR-33-5p targeted ABCA1 and CS

**Fig. 6:**
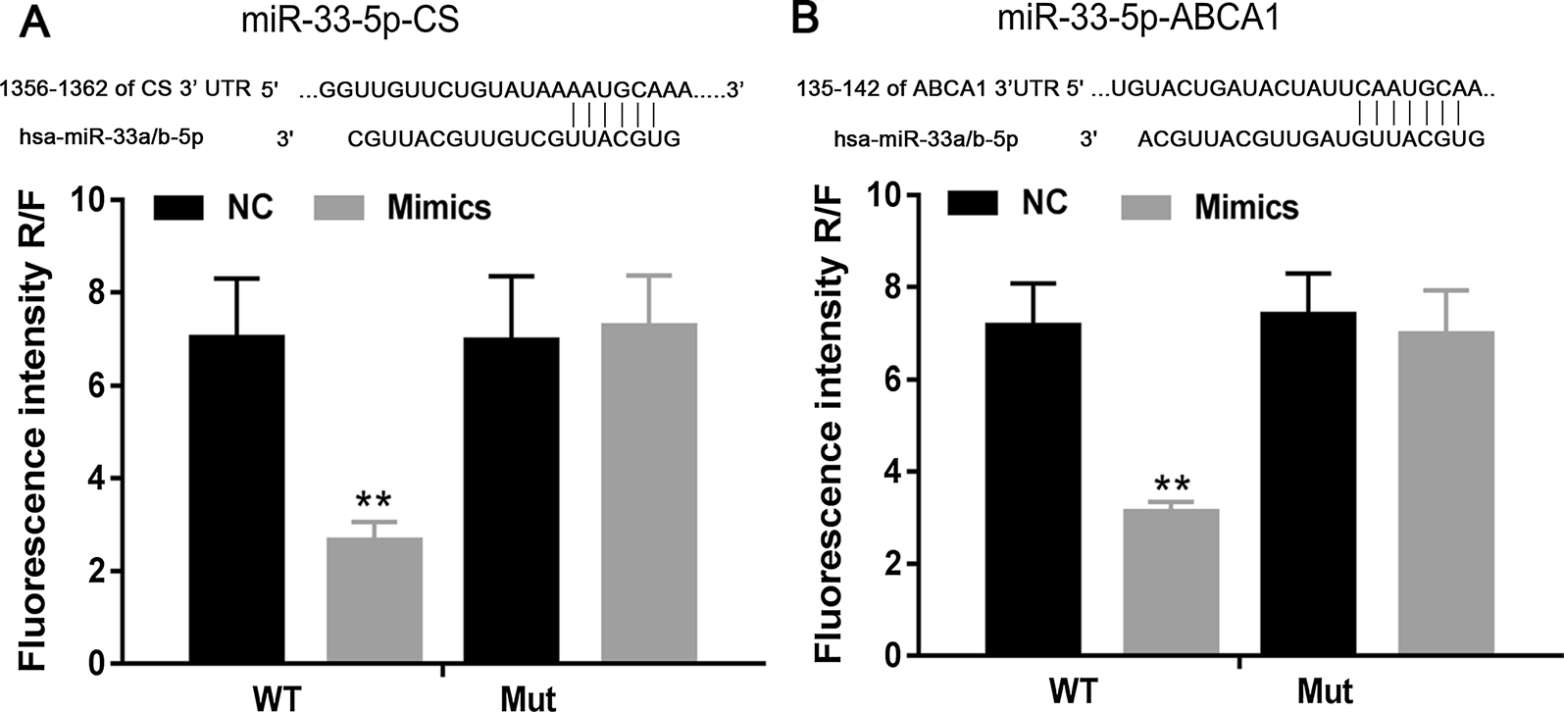
Dual-luciferase reporter assay results. **A** the prediction and determination of interaction between miR-33-5p and CS. **B** The predication and determination of interaction between miR-33-5p and ABCA1. NC, mimic negative control. WT, wild type. Mut, mutation. **p < 0.01 vs. control by one-way ANOVA followed by Tukey's post hoc test. The above experiments were repeated three times and the mean value was obtained

**Fig. 7:**
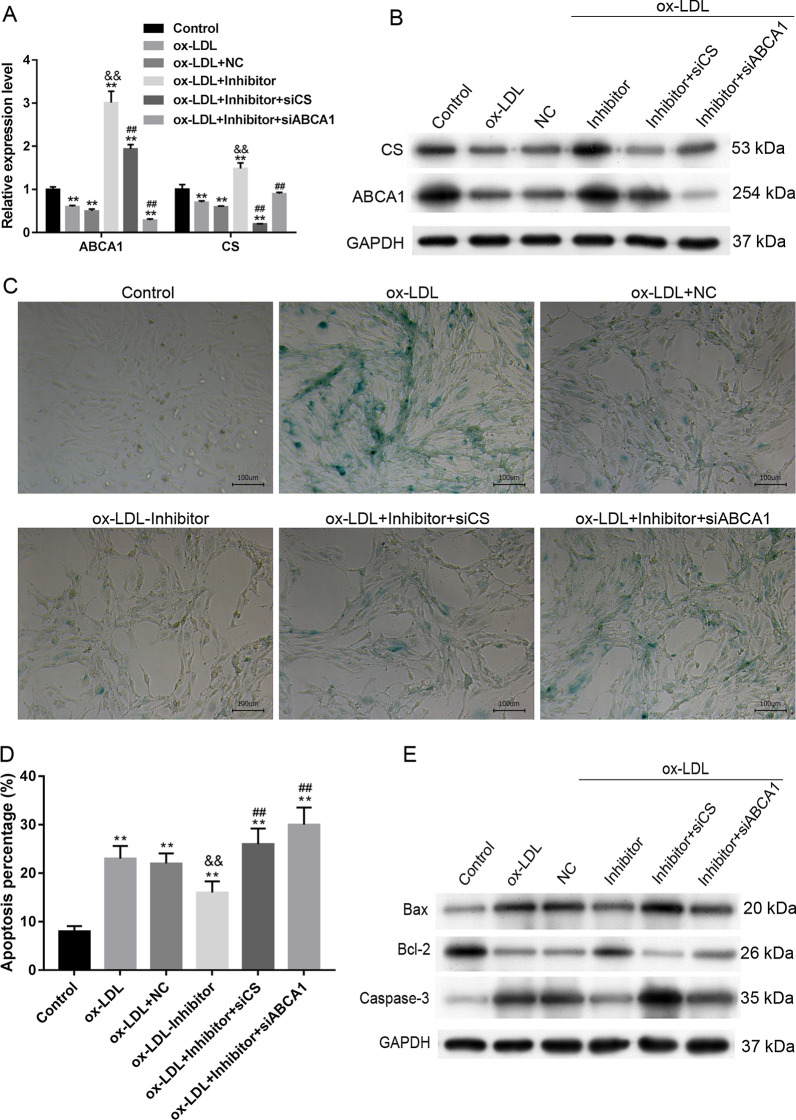
Antagonistic effect of CS and ABCA1 overexpression on miR-33-5p inhibition. **A**, **B** The levels of ABCA1 and CS mRNA and protein expression in response to transfections. **C** The levels of senescence-associated β-galactosidase (SA-β-gal) activity in VECs. **D** The percentages of apoptotic VECs. The repeated results were shown in Aditional file 2: Supplementary Figure 5. **E** The expression of apoptosis-related proteins. Ox-LDL, oxidized low-density lipoprotein. NC, overexpression negative control. The original bands of WB are presented in Additional file 1: [2A and 2B]. **p < 0.01 vs. control by one-way ANOVA followed by Tukey's post hoc test. ^&&^*p* < 0.01 vs. ox-LDL by one-way ANOVA followed by Tukey's post hoc test. ^##^*p* < 0.01 vs. OE-ABCA1 by the one-way ANOVA followed by Tukey's post hoc test. The above experiments were repeated three times and the mean value was obtained

The predicted target interactions of miR-33-5p with ABCA1 and CS were verified by dual-luciferase reporter assays (Fig. 6A, B). Further siRNA experiments showed that siCS and siABCA1 inhibited the miR-33-5p inhibitor-induced expression of ABCA1 and CS (Fig. 7A, B). Cell populations that were co-transfected with the miR-33-5p inhibitor plus siCS or siABCA1 had higher levels of SA-β-gal activity (Fig. 7C), higher percentages of apoptotic cells (Fig. 7D) and higher levels of Bax and Caspase 3 expression (Fig. 7E) when compared to cells transfected with the miR-33-5p inhibitor alone (*p* < 0.01). Bcl-2 expression was decreased by treatment with ox-LDLs and rescued by the miR-33-5p inhibitor, which was further inhibited by siCS and siABCA1 transfection (Fig. 7E). These experiments demonstrated the interaction of miR-33-5p with ABCA1 and CS.

### Knockdown of CS antagonized ABCA1-induced changes in VECs

**Fig. 8:**
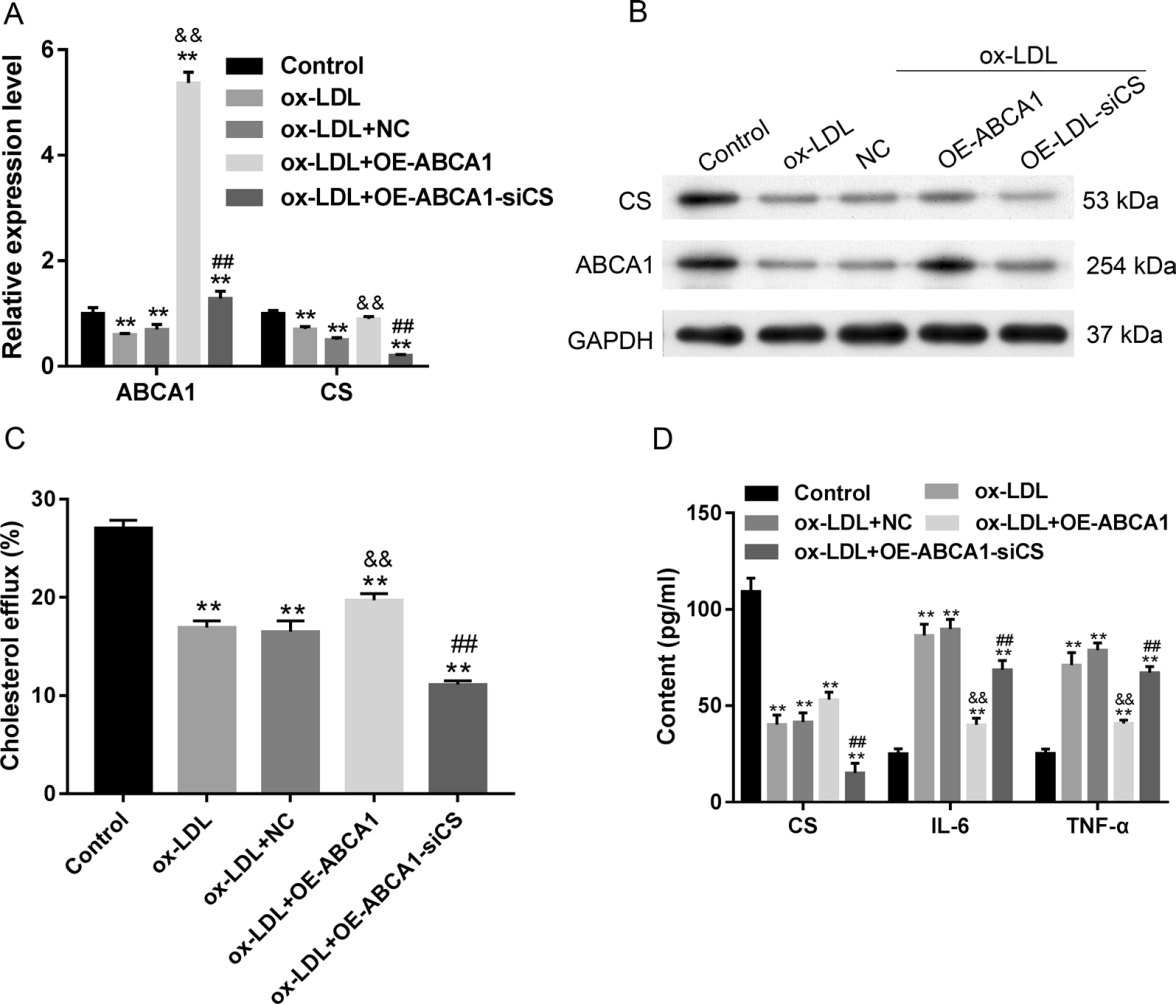
Antagonistic effect of CS overexpression on ABCA1 inhibition. **A**, **B** the levels of ABCA1 and CS mRNA and protein expression in response to transfections. The original bands of WB are presented in Additional file 1: [2C]. **C** the cellular cholesterol efflux from VECs. **D** the levels of cellular CS, IL-6, and TNF-α in VECs. Ox-LDL, oxidized low-density lipoprotein. NC, overexpression negative control. **p < 0.01 vs. control by one-way ANOVA followed by Tukey's post hoc test. ^&&^*p* < 0.01 vs. ox-LDL by one-way ANOVA followed by Tukey's post hoc test. ^##^*p* < 0.01 vs. OE-ABCA1 by one-way ANOVA followed by Tukey's post hoc test. The above experiments were repeated three times and the mean value was obtained

**Fig. 9:**
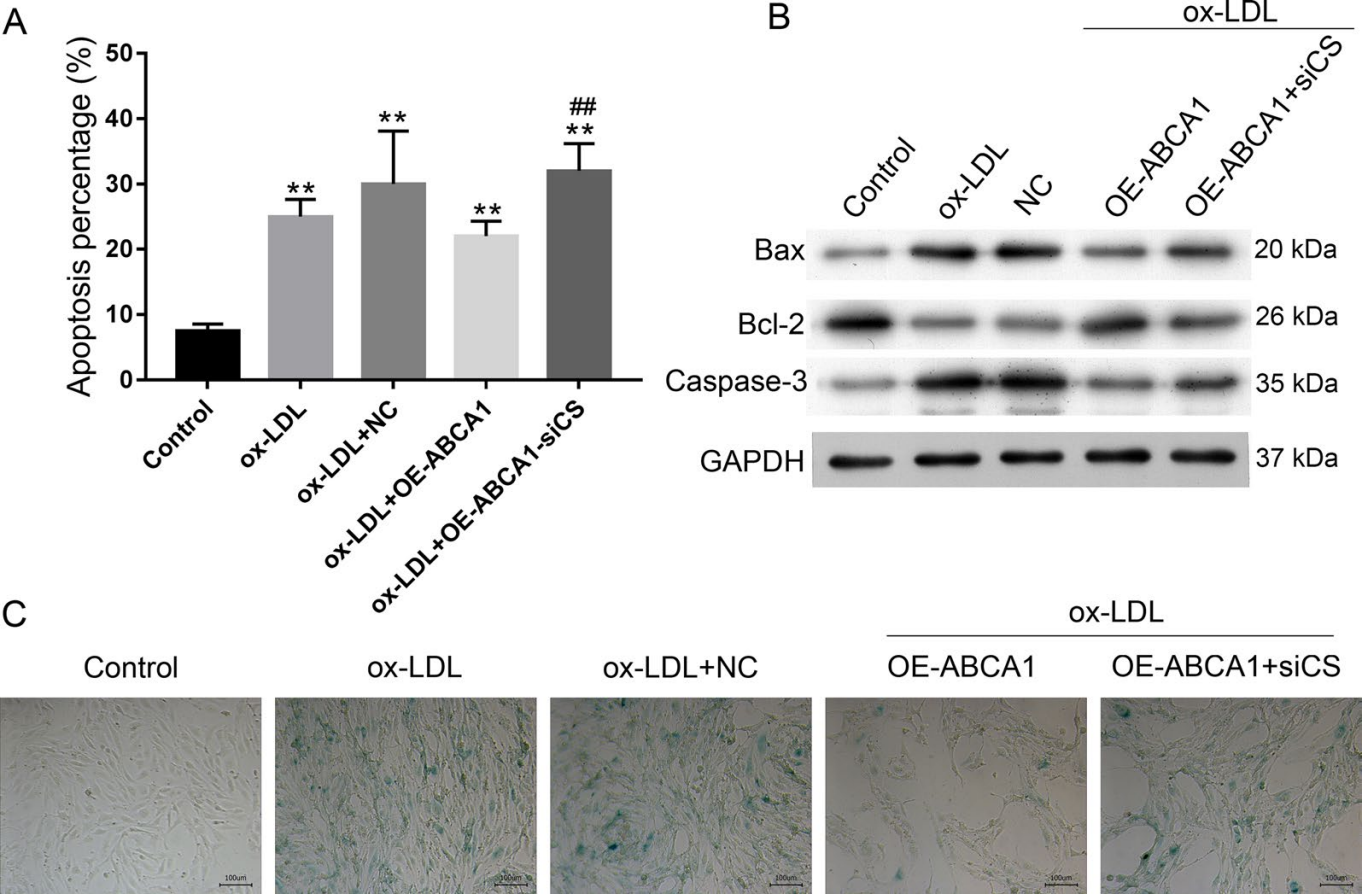
CS inhibition promoted VEC apoptosis and aging. **A** The percentages of apoptotic VECs. **B** The expression of apoptosis-related proteins. The original bands of WB are presented in Additional file 1: [2D]. **C** The levels of senescence-associated β-galactosidase (SA-β-gal) activity in VECs. The repeated results were shown in Aditional file 2: Supplementary Figure 6. Ox-LDL, oxidized low-density lipoprotein. NC, overexpression negative control. **p < 0.01 vs. control by one-way ANOVA followed by Tukey's post hoc test. ^##^*p* < 0.01 vs. OE-ABCA1 by one-way ANOVA followed by Tukey's post hoc test. The above experiments were repeated three times and the mean value was obtained

We confirmed that ABCA1 overexpression induced CS expression (Fig. 8A, B) and cholesterol efflux (Fig. 8C), and that ABCA1 inhibited inflammation (Fig. 8D), apoptosis (Fig. 9A), and SA-β-gal activity (Fig. 9C); furthermore, these changes could be rescued by siCS transfection. The decreases in Bax and Caspase 3 expression caused by ABCA1 overexpression were recovered by siCS; however, siCS decreased Bcl-2 expression (Fig. 9B). These results indicated that the ABCA1/CS axis was essential for inflammation, cholesterol efflux, apoptosis, and aging in VECs.

## Discussion

Low density lipoproteins (LDLs) are lipoprotein particles capable of carrying cholesterol into peripheral tissue cells, where it can be oxidized to form oxidized LDL. When the levels of LDLs, and especially ox-LDLs, become excessive, the cholesterol they carry will accumulate on the arterial wall and eventually cause arteriosclerosis. High density lipoproteins (HDLs) have received more attention as a preventive factor of atherosclerosis because they can export cholesterol and promote cholesterol metabolism. Furthermore, HDLs can help to atherosclerosis and protect against coronary heart disease. Many factors are known to be associated with cholesterol efflux, such as ABCA1 and miR-33-5p [14, 15]. Our present study demonstrated the role played by the miR-33-5p/ABCA1/CS axis in controlling cholesterol efflux in VECs. To the best of our knowledge, this is the first study to demonstrate the crucial role played by CS in cholesterol efflux in VECs.

ABCA1 is a direct target of miR-33-5p [14, 20]. Knockdown of miR-33-5p has been reported to decrease the levels of TC and TNF-α, and promote cholesterol efflux from macrophage-derived foam cells [20]. Our present study demonstrated the targeted interaction that occurs between ABCA1 and miR-33-5p. ABCA1 is crucial for removing excess lipids by initiating the RCT process and promoting cholesterol efflux to ApoA1 [18]. Our present study demonstrated that overexpression of ABCA1 forced by treatment with an miR-33-5p inhibitor or transfection with an overexpression plasmid promoted cholesterol efflux in VECs. In addition, ABCA1 overexpression and the miR-33-5p inhibitor prevented ox-LDL-induced inflammation, apoptosis, and aging in VECs. These data suggested the potential therapeutic effect of ABCA1 expression and miR-33-5p downregulation in modulating lipid metabolism by promoting cholesterol efflux.

CS is essential for cell growth [26]. Our study also demonstrated the similar crucial roles played by CS and ABCA1 in regulating cholesterol efflux, inflammation, and apoptosis in VECs. We first confirmed that CS was a direct target of miR-33-5p, and then showed that overexpression of CS impeded the ox-LDL-induced aging and apoptosis of VECs. In addition, out data showed that the ox-LDL-induced reductions in cholesterol efflux in VECs could be rescued by CS overexpression. In contrast, inhibition of CS and ABCA1 both promoted VEC apoptosis. These results were inconsistent those reported by Alhindi et al. [22], who showed that lower levels of CS activity were associated with increased levels of cleaved caspase-3 and lipotoxicity in C1C12 muscle cells. A low level of CS expression is associated with cell apoptosis and oxidative stress [27]. CS catalyzes the citric acid cycle in mitochondria and can serve as a biomarker of mitochondrial function in mammals [26]. In aging cells, mitochondrial function declines [26, 28]. Our present study demonstrated that siRNAs for both CS and ABCA1 could promote VEC aging and apoptosis. CS functions downstream of ABCA1 and was necessary for maintaining ABCA1-mediated cellular functions and cholesterol efflux in VECs. These findings suggested CS as a key and novel mediator of ABCA1-mediated cholesterol efflux.

Citrate plays important roles in innate immunity and inflammation [29]. It was reported that the natural auto-antibodies against CS (anti-CS IgM and IgG antibodies) were increased in rheumatoid arthritis (RA) patients treated with TNF inhibitors, certolizumab or etanercept therapy [30]. These antibodies might be associated with the clearance of apoptotic cells and restoration of immunological systems in the body [30]. Our study showed that inhibition of CS and ABCA1 increased the levels of IL-6 and TNF-α in VECs, while the overexpression of CS and ABCA1 reduced the levels IL-6 and TNF-α. In addition, suppression of CS increased the levels of ABCA1-reduced inflammatory cytokines (IL-6 and TNF-α) in VECs. These data suggested CS as a potent target for managing the inflammation-associated cholesterol efflux in VECs. However, the in vitro model used in our study has limitations, and an in vivo model such as apolipoprotein E-deficient (apoE(-/-)) hyperlipidemic mice or Abca1 binding site mutant (Abca1(BSM)) mice [31, 32] should to be used to further validate the role of the miR-33-5p/ABCA1/CS axis in cholesterol efflux. Moreover, citrate synthase activity has been shown to decrease after myocardial infarction and be partially regulated by MMP-9 [33]. Furthermore, as MMP-9 has been shown to cleave citrate synthase ex vivo, the change and involvement of MMP-9 in lipid metabolism disorders should also be further investigated, along with the role played by the miR-33-5p/ABCA1/CS axis.

## Conclusions

In conclusion, our present study demonstrated the crucial roles played by the miR-33-5p/ABCA1/CS axis in regulating cholesterol efflux, inflammation, apoptosis, and aging in VECs. We also found that CS had similar functions to ABCA1 in controlling VEC behavior and cholesterol efflux. CS and ABCA1 expression in VECs promoted cholesterol efflux and inhibited ox-LDL-induced apoptosis, inflammation, and aging. Our rescue experiments confirmed that CS acts downstream of ABCA1. The miR-33-5p/ABCA1/CS axis plays pivotal roles in lipid metabolism and the underlying mechanisms of lipid metabolism disorders such as atherosclerosis and CVDs.

## Supplementary Information

**Additional file 1.** The origin band of WB.

**Additional file 2.** Supplementary information of cells in multiple fileds.

## Data Availability

The datasets used and analysed during the current study are available from the corresponding author on reasonable request.

## Abbreviations

CVDs: Cardiovascular diseases
ABCA1: ATP-binding cassette (ABC) transporter A1
CS: Citrate synthase
VECs: Vascular endothelial cells
ox-LDL: Oxidative low-density lipoprotein cholesterol
IL-6: Interleukin-6
TNF-α: Tumor necrosis factor α
TC: Total cholesterol
SA-β-gal: Senescence-associated β-galactosidase
HDL: High-density lipoprotein cholesterol
HMGCR: 3-Hydroxy-3-methylglutaryl coenzyme A reductase\
RCT: Reverse cholesterol transport
ApoA1: Apolipoprotein A I
ELISA: Enzyme-linked immunosorbent assay
