# Supplementary material for: MicroRNA-33-5p inhibits cholesterol efflux in vascular endothelial cells by regulating citrate synthase and ATP-binding cassette transporter A1
Source: BMC Cardiovasc Disord. 2021 Sep 13;21:433. doi: 10.1186/s12872-021-02228-7 (PMC8438969; doi:10.1186/s12872-021-02228-7)
Supplement: Supplementary file 1 — Additional file 1. The origin band of WB. [file 12872_2021_2228_MOESM1_ESM.docx]

**MicroRNA-33-5p inhibits cholesterol efflux in vascular endothelial cells via regulating citrate synthase and ATP-binding cassette transporter A1**

Qiong Xie^1^, Jianqiang Peng^1^, Ying Guo^1^, Feng Li^2, *^

^1^ Department of Cardiology, Hunan Provincial People`s Hospital (The First Hospital Affiliated with Hunan Normal University), Changsha, Hunan 410005, PR China.

^2^ Departments of Cardiovascular Surgery, The Second Xiangya Hospital of Central South University, Changsha, Hunan 410011, PR China.

**^*^ Address correspondence to:** Dr Li, Departments of Cardiovascular Surgery, The Second Xiangya Hospital of Central South University, middle Ren-Min Road No. 139, Changsha, Hunan 410011, PR China. Email: muzicn@csu.edu.cn.

**The origin band of WB：**

Supplementary Figure 1：


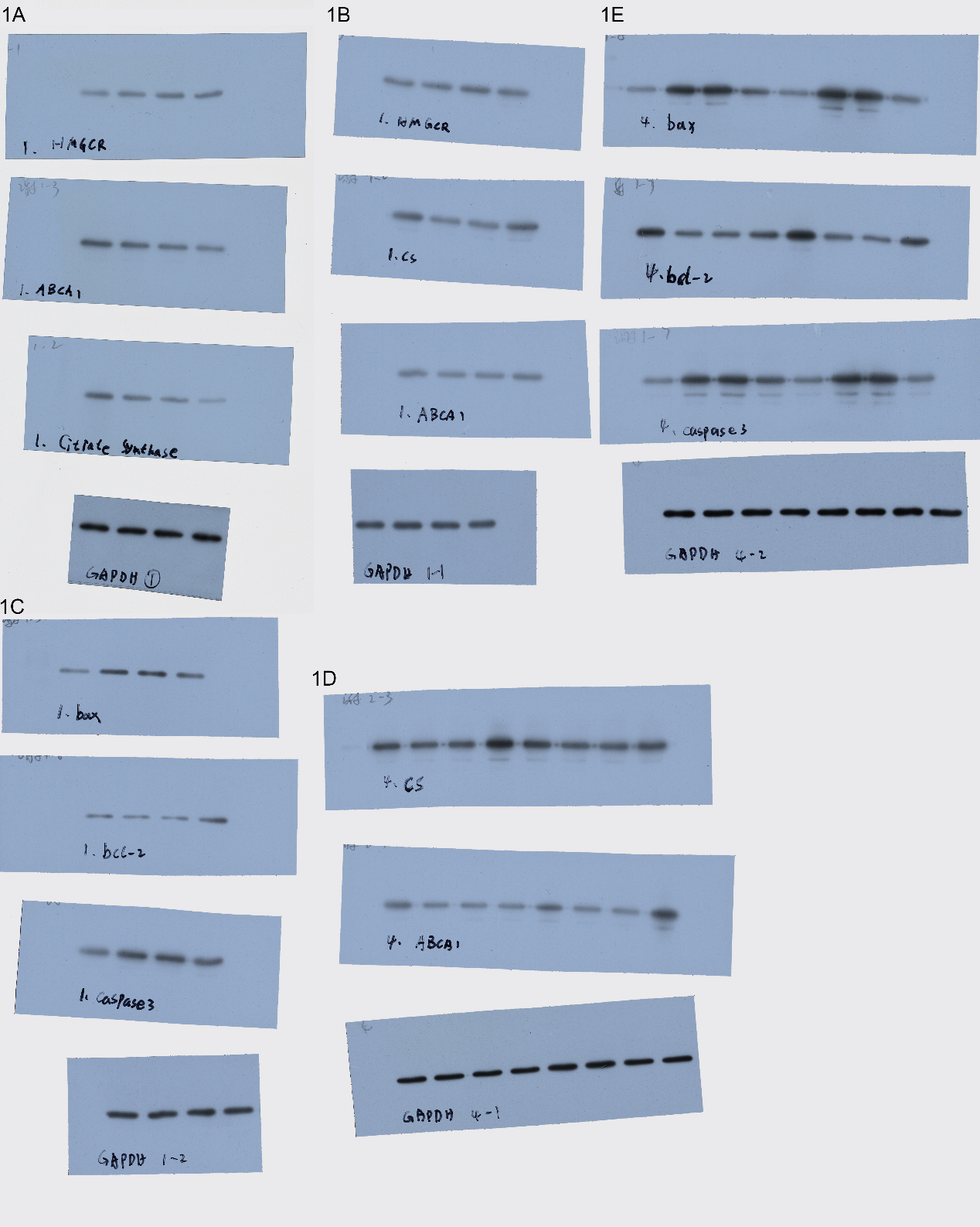


**Supplementary Figure1.** Original Western blot images of target proteins.The original band of WB in figure 1B (1A). The original band of figure WB in 2B (1B). The original band of figure WB in 3D (1C). The original band of figure WB in 4E (1D). The original band of figure WB in 5C (1E).

Supplementary Figure 2：


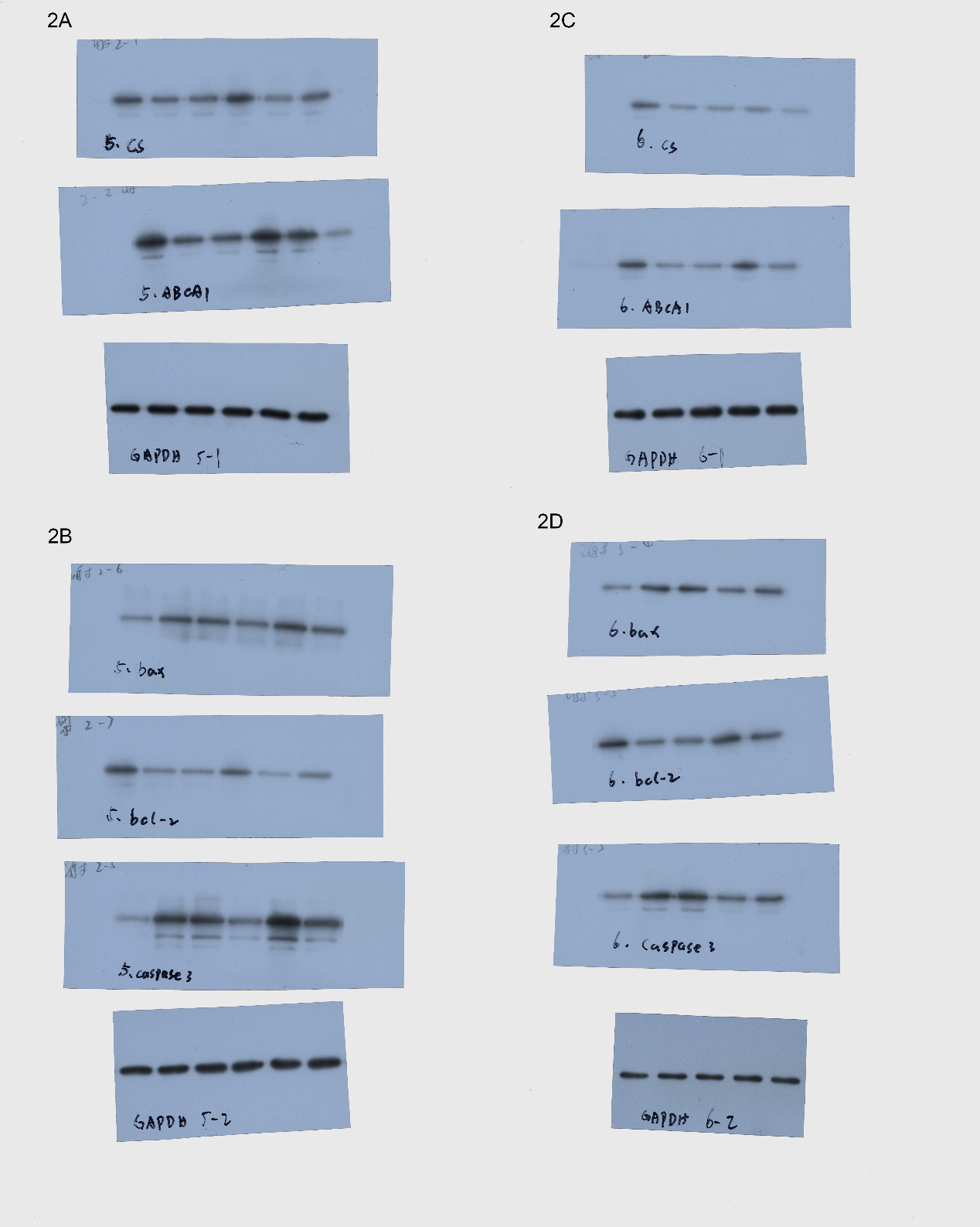


**Supplementary Figure2.** Original Western blot images of target proteins.The original band of WB in figure7B (2A). The original band of figure WB in 7E (2B). The original band of figure WB in 8B (2C). The original band of figure WB in 9B (2D).
