# Supplementary material for: MicroRNA-33-5p inhibits cholesterol efflux in vascular endothelial cells by regulating citrate synthase and ATP-binding cassette transporter A1
Source: BMC Cardiovasc Disord. 2021 Sep 13;21:433. doi: 10.1186/s12872-021-02228-7 (PMC8438969; doi:10.1186/s12872-021-02228-7)
Supplement: Supplementary file 2 — Additional file 2. Supplementary information of cells in multiple fileds. [file 12872_2021_2228_MOESM2_ESM.docx]

**MicroRNA-33-5p inhibits cholesterol efflux in vascular endothelial cells via regulating citrate synthase and ATP-binding cassette transporter A1**

Qiong Xie^1^, Jianqiang Peng^1^, Ying Guo^1^, Feng Li^2, *^

^1^ Department of Cardiology, Hunan Provincial People`s Hospital (The First Hospital Affiliated with Hunan Normal University), Changsha, Hunan 410005, PR China.

^2^ Departments of Cardiovascular Surgery, The Second Xiangya Hospital of Central South University, Changsha, Hunan 410011, PR China.

**^*^ Address correspondence to:** Dr Li, Departments of Cardiovascular Surgery, The Second Xiangya Hospital of Central South University, middle Ren-Min Road No. 139, Changsha, Hunan 410011, PR China. Email: muzicn@csu.edu.cn.

**Running title:** miR-33-5p/ABCA1/CS axis in cholesterol efflux

Supplementary Information of cells in multiple fields：

Supplementary Figure 3：


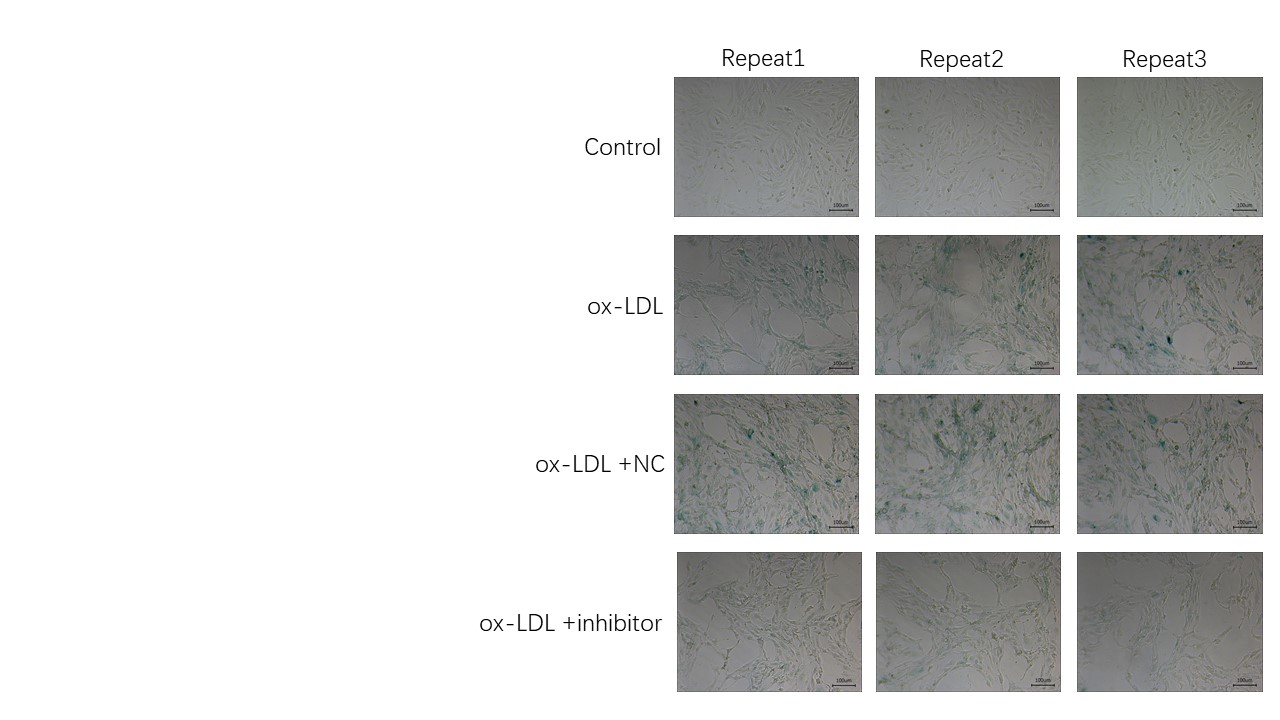


**Figure 3. Influence of miR-33-5p inhibition on apoptosis and aging of VECs. (**C) the inhibition of miR-33-5p on senescence-associated β-galactosidase (SA-β-gal) activity in VECs. The above experiments were repeated three times.

Supplementary Figure 4：


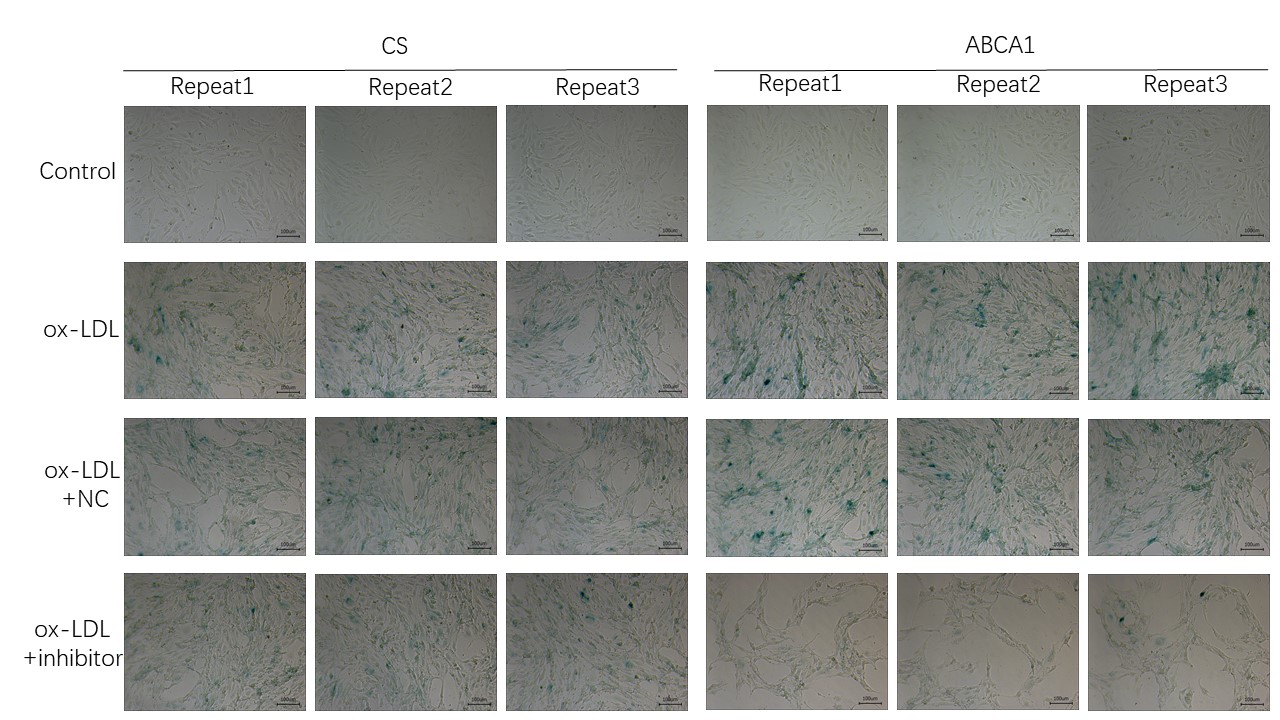


**Figure 5. Effect of CS and ABCA1 overexpression on cellular apoptosis and aging.** (A) the level of senescence-associated β-galactosidase (SA-β-gal) activity in VECs. The above experiments were repeated three times.

Supplementary Figure 5：


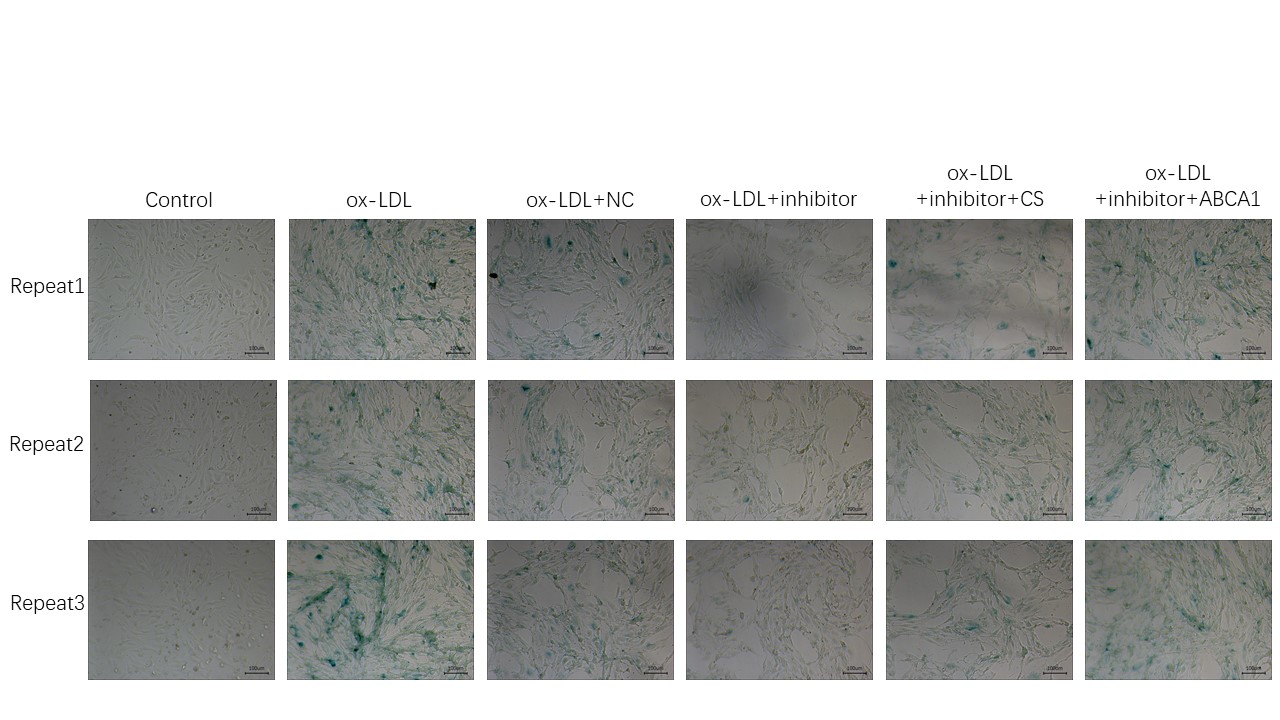


**Figure 7. Antagonism of CS and ABCA1 overexpression against miR-33-5p inhibition.** (C) the level of senescence-associated β-galactosidase (SA-β-gal) activity in VECs. The above experiments were repeated three times.

Supplementary Figure 6：


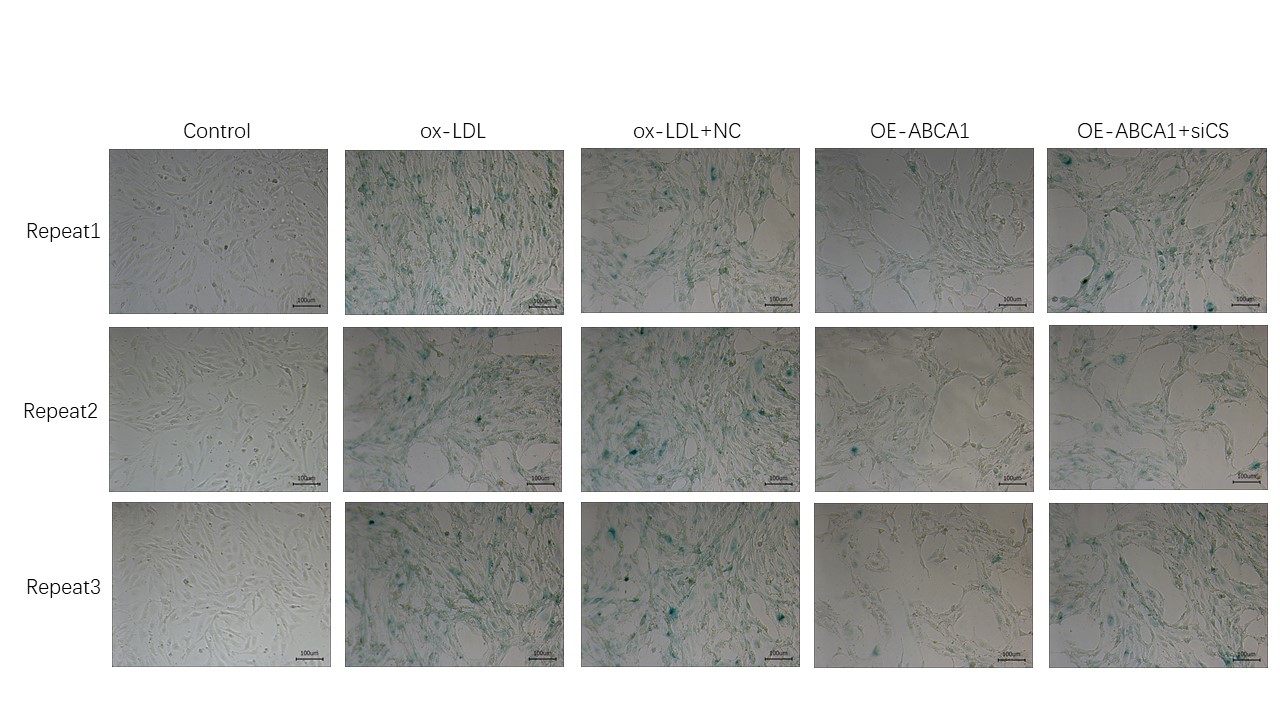


**Figure 9. CS inhibition promotes VECs apoptosis and aging.** (C) the level of senescence-associated β-galactosidase (SA-β-gal) activity in VECs. The above experiments were repeated three times.
